# Global health and big data: The WHO’s artificial intelligence guidance

**DOI:** 10.17159/sajs.2023/14725

**Published:** 2023-05-30

**Authors:** Kenneth W. Goodman, Sergio G. Litewka, Rohit Malpani, Sameer Pujari, Andreas A. Reis

**Affiliations:** 1 Institute for Bioethics and Health Policy, Miller School of Medicine, University of Miami, Miami, Florida, USA; 2 World Health Organization, Geneva, Switzerland

**Keywords:** artificial intelligence, big data, bioethics, global health, governance

## Introduction

Rarely in the history of science has a new tool or technology engendered the excitement, concern, and interest as artificial intelligence and machine learning in health and medicine. Although the Human Genome Project is a noteworthy antecedent in this regard, more lives will likely be touched by health information technology, including artificial intelligence (AI), than genetics – at least for the foreseeable future.

The world’s bioethics community has risen to the occasion with extraordinary thoughtfulness and, indeed, rapidity, as it seeks to keep pace with the ever-expanding uses of AI for health. Scholars on nearly every continent have turned or refocused their attention to challenges raised by the use of intelligent machines in clinical practice, public health, and biomedical research. This has led to a significant increase in the literature on AI and big data ethics over the past several years, including recommendations for appropriate use and users of a burgeoning technology.

Against this background, the World Health Organization (WHO), which for some two decades has supported a Global Network of Collaborating Centres for Bioethics^1^, organised a first WHO meeting on Ethics, Big Data and AI in 2017. This consultation, hosted by the University of Miami Miller School of Medicine’s Institute for Bioethics and Health Policy, was to identify the scope and range of ethical issues and questions related to big data and AI in health, in order to inform the work of WHO and to develop future principles and guidance for stakeholders.^2^

In 2020, after more than two years of consultations with Member States and many other stakeholders, the 73rd World Health Assembly adopted the ‘Global strategy on digital health 2020–2025’.^3^ The vision of the global strategy is to improve health for everyone, everywhere, by accelerating the development and adoption of appropriate, accessible, affordable, scalable, and sustainable person-centric digital health solutions to prevent, detect, and respond to epidemics and pandemics; to develop infrastructure and applications that enable countries to use health data to promote health and well-being; and to achieve the health-related Sustainable Development Goals and the ‘Triple Billion’ targets of WHO’s Thirteenth General Programme of Work, 2019–2023. The strategy is built on four strategic objectives:

1. To promote global collaboration and advance the transfer of knowledge on digital health.
2. To advance the implementation of national digital health strategies.
3. To strengthen governance for digital health at global, regional, and national levels.
4. To advocate people-centred health systems that are enabled by digital health.

These strategies are intended to provide guidance and coordination on global digital health transformation and to strengthen synergies between initiatives and stakeholders to improve health outcomes and mitigate associated risks at all levels.

Based on this previous work, WHO in 2020 committed to an 18-month process of guideline development, analysis of prior work and a comprehensive synthesis, leading to the 2021 publication of the WHO’s Guidance on *Ethics and Governance of Artificial Intelligence for Health*.^4^ The guidance was based upon the collective knowledge and insights of an international and multidisciplinary expert group from academia, government, industry, law, and non-governmental organisations, including human rights organisations, and represented all WHO regions. The report declares that^4^:

…for AI to have a beneficial impact on public health and medicine, ethical considerations and human rights must be placed at the centre of the design, development, and deployment of AI technologies for health. For AI to be used effectively for health, existing biases in healthcare services and systems based on race, ethnicity, age, and gender, that are encoded in data used to train algorithms, must be overcome. Governments will need to eliminate a pre-existing digital divide (or the uneven distribution of access) to the use of information and communication technologies. Such a digital divide not only limits use of AI in low- and middle-income countries but can also lead to the exclusion of populations in rich countries, whether based on gender, geography, culture, religion, language, or age.

The document reviews a variety of AI applications; salient laws, policies and principles; key ethical principles; ethical challenges; guidance for “building an ethical approach” to health AI; “liability regimes”; and several areas of governance – for example of data, intellectual property, and the private sector – that can assure that ethical principles can be effectively applied.

### Global data and AI context

Data are the fuel of artificial intelligence. Data from a vast range of sources are collected, stored, shared, and then analysed by AI systems, which are tuned or trained on very large data sets. There are, moreover, many data and information sources applicable to the use of AI for health, and they range across varied domains: mobile use and user location, clinical care, public health repositories and registries, biomedical research – as well as data which, while not explicitly about health, bears on people’s well-being. From finance and food to transportation and other social determinants of health, these and other domains all constitute and shape a vast digital ecosystem. Artificial Intelligence programs run on such records.

‘Data’ and ‘information’ are often and sometimes wrongly used synonymously. ‘Data’ has come, in many contexts, to refer to machine-readable or processable representations of facts. The binary code for ‘kidney disease’, for instance, is 01101011 01101001 01100100 01101110 01100101 01111001 00100000 01100100 01101001 01110011 01100101 01100001 01110011 01100101. Data can become information when rendered as facts humans can understand. A database might contain ones and zeroes, diagnostic references, or natural language expressions, for instance. In principle, all of these can be coded and so ‘de-identified’ or ‘pseudonymised’, or scrambled without a code and likely anonymised.

The ability to link or aggregate disparate data sets offers profound scientific opportunities, from improving diagnoses to guiding public health interventions to enhancing biomedical research. It also raises equally profound ethical issues. AI, or ‘knowledge discovery in databases’, mines these data sets in search of patterns. Such patterns could help clinicians prevent and treat disease but also, depending on the adequacy of security protocols and legal protections, expose individuals to confidentiality breaches. These patterns can help public health scientists identify disease trajectories and shape interventions to limit, say, pandemics – and they can foster stigma against some populations or population subgroups. In the opposite direction, to the extent that AI tools can improve clinical care and the health of populations, those individuals and populations without access to care and devices to improve it (those who exemplify the ‘digital divide’) are unlikely to benefit from the new technology. Generally, data applied to AI is biased towards the majority and may place a minority population – whether on the basis of race, gender, or age – at a disadvantage, with such biases enshrined in the AI.

Moreover, AI software can be difficult to explain and understand, and is sometimes or often not fully transparent; it is often biased; and it is frequently unclear who or what is responsible for oversight, maintaining standards, or ensuring safe use. This is in part the challenge of governance, some credible form of which is widely recognised as necessary if AI applications are to be trustworthy, trusted, and successfully used.

Against this background, the WHO guidance development group grappled with competing values, conflicting duties, and diverse stakeholder interests. It was essential to identify a set of core values that would undergird the final report and guide its conclusions and recommendations.

### Ethical principles

The WHO report reflects the trade-offs that should be considered to ensure that potential benefits of AI application to clinical practice, public health, or biomedical research do not outweigh the technology’s risks, while also assuring that certain core values and rights are fully protected. Most generally, it is uncontroversial to require that AI in health (and, indeed, in many other domains) be used fairly, avoid bias and discrimination, and promote equitable access. Healthcare systems can help achieve these ends by decreasing cost, ensuring diagnostic accuracy, and “storing and managing data [and] data collection via electronic health records, and exponential consumer data generation [creating] a data rich healthcare ecosystem”^5^.

Principles that should govern the development and use of big data and AI had already been enunciated by various organisations and countries. In fact, an analysis published in 2020 at the outset of the WHO guidance development process identified 36 sets of principles which either applied to the whole range of applications of AI or specific stakeholders/end-users (private sector, intergovernmental organisations, civil society, government, and multistakeholders).^6^ That and other initiatives point more broadly to the extraordinary amount of work devoted to establishing foundations for the ethically optimised use of AI tools. These initiatives may be regarded as a kind of international ethics “crowdsourcing”, the best antecedent for which is perhaps that of the Ethical, Legal, and Social Implications project that helped guide the Human Genome Project more than 30 years ago.^7^

The principles identified and agreed to by the WHO international expert group are the first specifically geared toward AI in health with international scope. The six principles endorsed by WHO are:

- protecting human autonomy
- promoting human well-being and safety and the public interest
- ensuring transparency, explainability and intelligibility
- fostering responsibility and accountability
- ensuring inclusiveness and equity
- promoting AI that is responsive and sustainable

The WHO’s experts intended these principles to be used as a basis for governments, programmers, companies, civil society, and intergovernmental organisations to adopt ethical approaches to guide appropriate use of AI for health. To be sure, any individual organisation might want to adapt or augment this or any set of principles and, indeed, the process of doing so should be regarded as an important exercise in ethics analysis, professional development, and community engagement.

### Ethical challenges

Principles alone do not provide guidance. They ‘govern’ conceptually and should inform debate surrounding practical questions and challenges. The first of these addressed by WHO was fundamental: should AI systems be used in the first place? Navigating between eager promotion and hyperbolic caution, the WHO report states that the benefits of AI systems can be realised only if they are unbiased, transparent, safe, and,

Even after an AI technology has been introduced into a health-care system, its impact should be evaluated continuously during its real-world use, as should the performance of an algorithm if it learns from data that are different from its training data. Impact assessments can also guide a decision on use of AI in an area of health before and after its introduction.^8^

Ethical challenges addressed by WHO’s work include^1^:

- Digital divide – It was clear that the growth and update of AI tools should not worsen disparities shaped by limited access, and that technology providers “should be required to provide infrastructure, services and programs that are interoperable” as countries narrow the divide.
- Data collection and use – From privacy to “function creep” and the commercialisation of personal data and information, the team debated the scope and limits of “appropriate use” and “appropriate users”.
- Data colonialism – At ground here, for instance, is the concern that high-income countries with “strict regulatory frameworks and data protection laws” might collect data from low- and middle-income countries that lack parallel data-protection laws.
- Accountability and responsibility – Basic ethical obligations related to standards, safety, and quality of AI systems rely on system developers, vendors, users, and their institutions to make plain and adhere to processes for ensuring best practices.
- Autonomous decision-making – The questions whether and to what extent an AI tool may operate without human control continue to be among the most interesting and challenging at the intersection of ethics and intelligent systems. Moreover, institutions must address the related questions of whether and to what extent patients and communities ought to be informed if self-governing machines are making medical or public health decisions.
- Bias and discrimination – That training sets introduce racial and other biases into AI systems remains a source of deep disquiet among scholars and advocates. Awareness and a plenary attention to mitigation is essential if future AI tools are to enjoy the trust of the communities they purport to serve and not exacerbate existing biases that undermine healthcare provision and patient outcomes.
- Safety and cybersecurity – Among key findings here is that safety and security issues might arise even after a thorough review before a system’s implementation. This underscores the need for ongoing vigilance.
- Labour and employment – AI adoption might have a deleterious effect on clinicians’ professional development and engender skill degradation and, indeed, good systems might even replace traditional humans through various forms of automation.
- Commercialisation – Although markets can drive innovation, they can also corrupt the environments they shape. A concern raised by the expert team: “When most data, health analytics and algorithms are managed by large technology companies, it will be increasingly likely that those companies will govern decisions that should be taken by individuals, societies and governments…”
- Climate change – Some AI applications generate non-trivial emissions of greenhouse gases and have other effects on the environment. The WHO working group calls for “stringent oversight by governments and good governance”.

The process to develop the guidance document revealed the rich scope of AI ethical issues and challenges faced by the world’s health community, as well, significantly, as the extraordinary effort by the informatics and ethics scholars to address them. Indeed, the task of analysing and synthesising the many previous and ongoing efforts to foster ethical and trustworthy AI – and doing so for an international community – was an opportunity to identify the most compelling arguments for good practice, as well as those approaches most likely to succeed. An overarching goal was to encourage consensus in a complex and fraught environment.

### Governance

Good governance requires more than carefully vetted and balanced values. In parallel to the appropriate and adequate oversight of AI systems, the WHO working group addressed issues of data control and sharing, data sovereignty, transparency, valid consent and its breadth or scope, benefit sharing, and the potential role of federated data. An exemplary governance scheme must also encompass accountability and responsibility. Two overarching governance questions need to be addressed: what exactly should be governed and who or what should do it? According to the guidance document^4^:

Governance in health covers a range of steering and rule-making functions of governments and other decision-makers, including international health agencies, for the achievement of national health policy objectives conducive to universal health coverage. Governance is also a political process that involves balancing competing influences and demands.

The rapid and broad growth of AI research, development, and adoption embeds numerous points and processes to monitor and influence.

The software development lifecycle is already in many cases vetted for reliability and quality, albeit not explicitly for ethics. Likewise, the creation, maintenance, and use of databases used for training AI algorithms. The question of which points and processes to oversee or scrutinise will likely be best answered after a thorough review of which oversight strategies are found most effective in achieving the goal of fair and trustworthy systems. This is in part an empirical question.

As to the question of what entities should exercise a governance function, the most apt approach will be multifaceted. This means that there might be a role for software developers themselves; their commercial, academic, and government employers; institutions that use the systems; professional societies; perhaps even a kind of lay oversight, a regulatory version of ‘citizen science’. There is also a need for legislative action to compel testing, evaluation, and adherence to best practices, and a regulatory apparatus that can put such laws into good practice. WHO is currently developing a separate guidance document that examines regulatory considerations that governments may adopt. There are already ample precedents for such regulatory supervision in the form of data privacy laws in individual states (e.g. South Africa’s *Protection of Personal Information Act* and the *Health Insurance Portability and Accountability Act* in the USA) and their federations (the European Union’s General Data Protection Regulation). As is the case in many other areas of health care, civil society, patients, and communities that are most directly affected by the deployment of such technologies must have adequate means to influence the development and use of AI. Thus, the WHO guidance document recommends^4^:

Patients, community organizations and civil society should be able to hold governments and companies to account, to participate in the design of technologies and rules, to develop new standards and approaches and to demand and seek transparency to meet their own needs as well as those of their communities and health systems.

## Conclusion

WHO and several other organisations have issued normative frameworks on the ethical development and use of AI for health. Now more efforts are needed to ensure that these international norms are taken up by the various stakeholders (from governments to industry) and implemented in daily practice. Specific tools need to be developed (for programmers to actually implement ‘ethics by design’ in their work; for governments to address the ethical challenges in their laws and regulations; etc.). Technology and knowledge transfer need to be promoted alongside investments to overcome an enduring digital divide. The effort to forge the first global guidelines to meet ethical challenges raised by this exciting new technology is both an affirmation of shared values and an opportunity to ensure appropriate use of this technology.

## Significance:

The growth and adoption of artificial intelligence tools and systems has the potential to transform health and wellness, even as this expansion raises challenging ethical issues, including data and privacy protection, appropriate uses and users, human rights concerns, and inequitable access. The WHO in 2020 committed to an 18-month process of guideline development, leading to the 2021 publication of the WHO’s Guidance on *Ethics and Governance of Artificial Intelligence for Health*. The document identifies salient ethical principles, assesses a range of ethical issues and challenges, addresses governance strategies, and offers recommendations; it is apparently the first to offer global guidance.
